# The Impact of Financial and Non-Financial Work Incentives on the Safety Behavior of Heavy Truck Drivers

**DOI:** 10.3390/ijerph18052759

**Published:** 2021-03-09

**Authors:** Sebastjan Škerlič, Vanja Erčulj

**Affiliations:** 1 Faculty of Maritime Studies and Transport, University of Ljubljana, Pot pomorščakov 4, 6320 Portorož, Slovenia; 2 Faculty of Criminal Justice and Security, University of Maribor, Kotnikova ulica 8, 1000 Ljubljana, Slovenia; vanja.erculj@fvv.uni-mb.si

**Keywords:** financial incentives, non-financial incentives, compensation, truck drivers, safety, safety behavior

## Abstract

The goal of the research is to determine how compensation affects the safety behavior of truck drivers and consequently the frequency of traffic accidents. For this purpose, a survey was conducted on a sample of 220 truck drivers in international road transport in the EU, where the results of the Structural Equation Model (SEM) show that in the current state of the transport sector, financial and non-financial incentives have a positive impact on the work and safety behavior of drivers. Financial incentives also have an impact on drivers’ increased perception of their driving ability, while moving violations continue to have a major impact on the number of accidents. The proposed improvements enable decision-makers at the highest level to adopt legal solutions to help manage the issues that have been affecting the industry from a work, social and safety point of view for the past several years. The results of the research therefore represent an important guideline for improvements to the legislature as well as in the systematization of truck driver compensation within companies.

## 1. Introduction

A truck driver’s job is to transport cargo for the owners of the goods transported (shippers and recipients) or for those who pay for their movement. Since contracts between cargo owners specify the product, quantity, and distance, in most cases and in most parts of the world, transport companies pay drivers on the basis of the kilometers driven or the percentage of income charged for the transport of goods [1]. In the EU, this method of compensation is officially prohibited, as it is contrary to Article 10 of Regulation (EC) no. 561/2006. However, what we have observed in practice is that transport companies do not adhere to these rules to the same extent across all EU countries [2]. Another issue stems from the fact that in the EU, truck driver compensation is subject to different interpretations from country to country. Research has also shown that the largest portion of a driver’s wage is variable and based on productivity-related payment practices, despite it being prohibited: pay per kilometer, pay per trip or payment linked to a percentage of the revenue received for the carriage of the goods [3].

Faulkiner and Belzer [4] and Belzer and Sedo [5] who have thoroughly researched this subject in recent years highlight the negative impact of productivity-based compensation on truck drivers’ health and road safety. However, the authors also point out that truck drivers with better wages are also safer drivers, which results in fewer accidents. They highlight the importance of the efficiency wage theory, which is based on the assumption that offering higher compensation attracts higher-quality applicants, partly because higher compensation is a signal for higher-quality jobs. Authors point out that the higher the compensation, the more motivated the drivers are to work for employers, the less likely they are to shirk responsibilities and the greater their incentive to operate trucks safely. However, it is entirely possible to argue that drivers subconsciously have no intention to cause an accident, notwithstanding the fact that they are being compensated less than others. In contrast with previous studies, we believe that, in certain situations, a satisfied or financially better-compensated truck driver might even be more inclined to exceed driving time limits and shorten daily and weekly rest periods. Ultimately, this can all lead to reduced road safety.

This study aims to draw attention to the fact that the issues surrounding truck driver compensation are somewhat complex, but consistent in nature and require an even more in-depth examination. The latest research results by Kudo and Belzer [1], where they analyzed how the financial compensation of truck drivers in the U.S. affects their safety performance, also suggest a partial departure from previous findings by other researchers. When determining whether transport companies should offer higher compensation in order to attract drivers with higher human capital and thus motivate them to operate safely, the results did not produce statistically significant differences. The impact of financial compensation on safety was also specifically explored by Belzer and Sedo [5], who highlighted the importance of reaching earnings targets. They emphasized that a higher mileage rate means higher revenue for drivers, which means that they reach their target earnings in a shorter time. As a result, they can drive fewer kilometers, work fewer hours, and have no need to violate driving regulations to reach their target earnings, which in turn leads to greater safety. However, the question is whether it is even possible for truck drivers to manage their target earnings in an industry characterized by employer pressures to maximize the number of trips, customer pressure to reduce freight rates, and pressures to deliver goods faster. It is precisely because all of these factors that we cannot claim with absolute certainty that drivers who receive better financial compensation and who reach the expected target earnings will also be more safety-oriented. The factors described therefore raise the following research questions:

What is the actual impact of financial incentives on moving violations committed by truck drivers and how does this affect road safety?

Could solutions for ensuring safe working conditions in the transport sector be linked to better non-financial compensation of truck drivers?

Does better compensation really enhance the driving ability of truck drivers and how does this affect their safety behavior in traffic?

To answer these research questions, a study was conducted on a sample of 220 drivers in international road transport within the EU. The goal of the research is to determine how financial and non-financial work incentives affect the safety behavior of truck drivers and consequently the frequency of traffic accidents. Thus, the Structural Equation Model (SEM) developed leads to three distinct research directions. The first one is the analysis of the impact of financial incentives on the safety behavior of truck drivers, which has been the subject of wide-ranging academic, industry, and governmental debates in different parts of the world. The second direction is that of the analysis of the impact of non-financial incentives on the safety behavior of truck drivers, which is a globally under-researched topic that remains not fully understood. The third direction is the analysis of the impact of both types of incentives (financial and non-financial) on the driving abilities of truck drivers and consequently their safety behavior.

In the USA, the subject discussed has been thoroughly researched in recent years. Authors Belzer and Sedo [5] found that financial incentives affect the drivers by reducing the number of hours they spend working, as they reach their expected earnings more quickly, which in turn leads to greater safety. Faulkiner, M.R.; Belzer [4] also came to similar conclusions by pointing out that better compensation attracts better quality truck drivers, leading to safer truck management. The study conducted by Kuda, T. and Belzer [1] is also relevant, as they searched for a link between truck driver compensation and safety performance. The study is therefore the first of its kind in the EU, meaning that its results represent a scientific novelty. The results are also relevant on a global level, as they show the impact of non-financial compensation on the safety behavior of truck drivers. Although the results are difficult to compare due to differences in labor laws in the transport industries in the US, Australia, Canada, and the rest of the world, we made a comparison in the Discussion part of the study. The study thus enables a comparison with other truck driver compensation systems and proposes improvements in the transport sector, which is subject to fierce competitiveness and constant pressure to achieve work efficiency throughout the world.

The results obtained therefore add to the current field of research on truck drivers’ compensation, both financial and non-financial. They represent an important instrument for guiding in improving the compensation of drivers in transport companies and an important source of information for legislative and government decision-makers in setting new rules of operation in the transport sector.

## 2. Literature Review and Development of a Structural Research Model

The negative impact of productivity-based compensation on drivers’ health, the quality of transport services and road safety has been highlighted in recent years by Belzer and Sedo [5], Belzer [6], and Faulkiner and Belzer, 2019 [4]. However, at the same time point out that truck drivers with better wages are also safer drivers, which results in fewer accidents. However, it is entirely possible to argue that drivers subconsciously have no intention to cause an accident, notwithstanding the fact that they are being compensated less than others. In contrast with previous studies, we believe that, in certain situations, a satisfied or financially better-compensated truck driver might even be more inclined to exceed driving time limits and shorten daily and weekly rest periods [7]. The most recent results of the study conducted by Kudo and Belzer [1] confirm this, as they indicate a partial departure from previous findings. Similarly, the target earnings hypothesis developed by Belzer and Sedo [5] does not guarantee better safety results in the transport sector. The hypothesis states that a higher mileage rate means a higher income for the drivers, which means that they reach their target earnings in a shorter time. As a result, the drivers can drive fewer kilometers, work fewer hours, and have no need to violate driving regulations to reach their target earnings. This, in turn, leads to greater security. However, the question remains whether it is even possible for drivers to manage their target earnings in an industry characterized by pressures from employers to maximize work efficiency.

It is precisely because of all of these factors that we cannot claim with absolute certainty that drivers who receive better financial compensation and who reach the expected target earnings will also be more safety-oriented. For the purpose of elucidating this issue, the following assumptions and hypotheses that will contribute to the development of the Structural Equation Model were made:

The issues surrounding the compensation of truck drivers are further expanded when framed within the adaptation theory [8,9], as truck drivers do have a choice. They may opt for short-distance driving jobs, which pay at an hourly rate, but their earnings will be lower [5]. However, as long-distance truck driving jobs are known to often involve overtime, drivers are accustomed to working overtime, accepting a productivity-based payment method [5], and adapting to the situation [9]. In fact, research has also shown that wages in the transport sector motivate drivers to work longer hours [10,11], which can encourage drivers to drive overtime, shorten rest periods (daily and weekly), and manipulate tachographs and driver cards [12]. However, this type of behavior may be the result by pressures from employers to make as many journeys as possible [6,7,8,9,10,11,12,13] and the conscious, adapted behavior of truck drivers to maximize earnings [8,9]. Thus, our first hypothesis is as follows:

**Hypothesis** **1** **(H1).** *Financial incentives affect the propensity to exceed driving time (H1a) and shorten daily (H1b) and weekly rest (H1c).*

Although many studies [14,15] put a lot of emphasis on the fact that drivers are most attracted to and motivated by high wages and additional financial incentives, a study by Prockl et al. [16] argues that money cannot buy job satisfaction. According to their study, non-financial job properties include work time allocation as agreed, a pleasant work environment, the management team and style, and career development opportunities. This adds relevance to non-financial incentives, as the results of a survey on the subjective well-being of employees in the transport sector are well below average in terms of life satisfaction and job satisfaction [17]. Additionally, Stewart et al. [18] have found that low levels of well-being among workers can have a variety of detrimental effects, including reduced work productivity. The impact of non-financial incentives is undeniable, as they can affect the work motivation of truck drivers and therefore represent an important alternative to financial compensation. However, in this case, there may be a bilateral interest in the employer–employee relationship. The transport sector is mostly geared towards demanding greater productivity from truck drivers [19,20,21,22], which is why some employers offer a range of different non-financial incentives. A truck driver will put in more work in order to stay with the current employer, as offering non-financial job properties increases loyalty [16]. This leads to our next hypothesis:

**Hypothesis** **2** **(H2).** *Non-financial incentives affect the propensity to exceed driving time (H2a) and shorten daily (H2b) and weekly rest (H2c).*

Kudo and Belzer [1] point out that in the transport sector, greater financial incentives attract more productive job seekers. As other researchers have also been pointing out for many years, workers with greater human capital can be more productive and possess high-level work skills, allowing companies to pay them higher financial compensation [23]. Rodriguez et al. [24] also point out that financial incentives are necessary to recruit individuals who possess highly valued qualities. Therefore, a conclusion can be drawn that drivers who meet these criteria will be able to effectively balance their work obligations during the week with adequate rest and other activities that contribute to less fatigue and better driving abilities. This leads to our next hypothesis:

**Hypothesis** **3** **(H3).** *Financial incentives affect the driver’s ability to operate the truck.*

Belzer and Sedo [5] emphasize that truck drivers are increasingly considering employment as a kind of package of benefits that are not just financial in nature and come with fewer work hours and a regulated work environment. Drivers who drive fewer kilometers and work fewer hours are less likely to switch jobs. Prockl et al. [16] point out that companies that can offer a regulated work environment, a fair management team, and career development opportunities will also gain truck drivers with high human capital who will be loyal to the employer. Drivers who fall into this category are also more productive and can possess high-level work skills [23] that allow them to effectively manage their work commitments throughout the week. Thus, our next hypothesis is as follows:

**Hypothesis** **4** **(H4).** *Non-financial incentives affect the driver’s ability to operate the truck.*

Although Kudo and Belzer [1] have found that 80 per cent of drivers have no moving violations, Viscelli [25] points out that even with electronic logbooks, drivers systematically violate the HOS (Hours of Service) maximum labor time regulation because non-driving labor time requires self-reporting and drivers’ economic incentives run counter to long-term safety and health policy. Murphy et al. [26,27] also point out the possibility of violations by drivers, as they found that the positive impact of safety climate on truck drivers’ driving safety behavior was slightly weakened as the number of years spent as a truck driver increased. Through their work experience as truck drivers, which includes their exposure to social and external systems, both in a narrow (e.g., specific maintenance regulations) and wide sense (e.g., national norms or culture), drivers have developed their own beliefs that help them do their job with efficiency. This also means that their capacity to determine what is right and what is wrong is based on their perception of any given work situation and a reflection of many years of experience. Therefore, we cannot exclude the possibility that more capable and experienced drivers might violate the rules in certain situations. Thus, our next hypothesis is as follows:

**Hypothesis** **5** **(H5).** *The greater the drivers’ ability to operate the truck, the greater the propensity to exceed driving time (H5a) and shorten daily (H5b) and weekly rest (H5c).*

Regulation (EC) 561/2006 [7] lays down rules on driving times, breaks and rest periods for drivers engaged in the carriage of goods and passengers by road in the EU. The situation is similar in the United States, where hours of service regulations (HOS) limit driving time and impose rest periods for commercial vehicles driving long distances [28]. The purpose of the regulations is to reduce truck driver fatigue, improve working conditions in the transport sector, and reduce traffic accidents [29,30,31]. Williams and Monaco point out [32] that truck drivers who violate HOS rules are more likely to have accidents. Viscelli [25] cites the possibility of systematic HOS violations, as drivers are only required to report the time they do not spend driving. The author draws attention to the issue that the current economic incentives in the transport sector run counter to long-term traffic safety and driver health. Violations in the transport sector can therefore have serious consequences for the safety of all road users, leading to the last hypothesis, which is as follows:

**Hypothesis** **6** **(H6).** *Exceeding driving time (H6a) and shortening daily (H6b) and weekly rest (H6c) have an impact on drivers’ accidents.*

**Figure 1:**
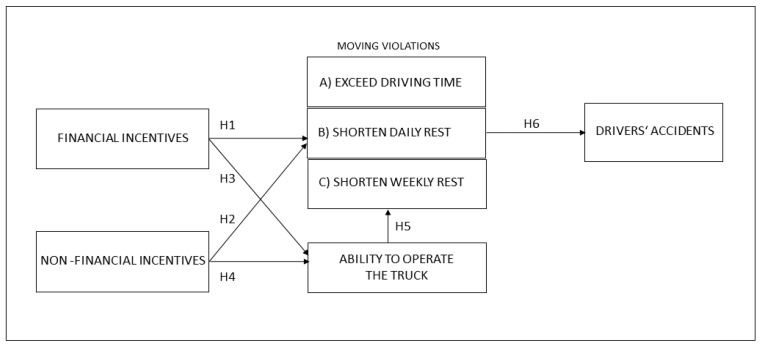
Structural Equation Model.

Based on the hypotheses proposed, a Structural Equation Model (Figure 1) has been developed, the first phase of which consists in determining the impact of financial incentives on moving violations by truck drivers and how this affects road safety. The next phase of the research aims to determine whether solutions for ensuring safe working conditions in the transport sector could be linked to non-financial compensation of truck drivers. In the final phase of the research, the impact of both types of incentives on the driving abilities of truck drivers and the associated safety behavior will be analyzed.

## 3. Methods

A total of 731 heavy-duty truck drivers in international road freight transport were invited to participate in the survey, of which 220 drivers accepted the invitation. An online questionnaire was prepared, which guaranteed anonymity to the respondents and sent via SMS to the mobile phone of each driver who had previously accepted to participate in the survey. Due to the high frequency of truck drivers, the truck terminals in the Port of Trieste (Italy) and the Port of Koper (Slovenia) were chosen as locations, as well as, in part, a couple of large truck stops. The two ports supply goods to parts of western Italy, parts of southern Germany, Austria, Slovenia, Hungary, the Czech Republic, Slovakia, and parts of southern Poland. Due to the international nature of the survey, answers were provided in English, German, Italian, Slovenian, Croatian, and Serbian. In all, 96 truck of the drivers were from Slovenia, 92 from Serbia, 37 from Croatia, 22 from Italy, and 2 from Austria.

Prior to the official submission of the questionnaire to the drivers, the questions were reviewed by five heavy truck drivers who were not subsequently included in the study. The questionnaire was also reviewed by two directors of major international transport companies. The survey was conducted from 15 July 2020 to 15 September 2020.

The questionnaire was developed as follows: Non-financial work incentives were included based on the definitions in the study by Prockl et al. [16]. Part of the financial incentives were included based on the definitions in the study by Kudo and Belzer [1] and partly based on the study by Prockl et al. [16]. Regulatory issues were included based on EC Regulation 561/2006 [17], which defines the rules on driving time, breaks and rest periods for drivers carrying goods and passengers by road. The classification of road accidents is based on the Internal Code of Traffic Accidents, V1 [33].

### 3.1. Participants

The final sample included n = 220 truck drivers. One woman participated in the study, while other participants were males. The average age (SD) of participants was 40.8 (10.7) years. Approximately one third (37.7%) had more than high school education. The average (SD) number of years working as a truck driver was 13.7 (10.1) years. On average (SD), they are employed at the current employer for 5 (5.1) years. The drivers drive an average of 2557.10 km per week. In the last month, 73% of the drivers did not exceed the daily driving limit; 70% of the drivers shorten their daily rest period in accordance with EC Regulation 561/2006 and 77% of the drivers shorten their weekly rest period in accordance with EC Regulation 561/2006.

### 3.2. Measures

Financial incentives (Cronbach’s α = 0.76) was measured by four indicators: “The salary is sufficient for covering living expenses”, “Working on a free weekend outside home results in additional financial incentives”, “Working on holidays results in additional financial incentives”, and “Spending less fuel than normative results in additional financial incentives.”

Non-financial incentives (Cronbach’s α = 0.89) was measured by six items: “The communication with my superior is correct”, “Drivers in the company help each other”, “In the company, they praise me when I deserve it”, “When I started working, the company helped me with inclusion into the working process”, “In the company, they are ready to help me if I need something”, and “The company superiors respect me.” Cronbach’s α equals 0.82.

Truck management (Cronbach’s α = 0.74) was measured by three items: “When the daily time of driving 9/10 h is ending, I still feel capable of driving”, “When the daily rest time of 11 h is ending, I feel able to start driving sooner”, and “On Fridays, at the end of the workweek, I feel rested.”

Car accidents (Cronbach’s α = 0.67) was measured by three items: “Fill in the number of accidents with financial damage”, “Fill in the number of accidents where one or more people suffered minor injuries”, and “Fill in the number of accidents where one or more people suffered major injuries”.

Exceeding daily driving limit was a single item measure “Fill in the number of times you exceeded the daily driving limit of 9/10 h in the last month.”

Shortening daily rest limit was a single-item measure. “Fill in the number of times per week you usually shorten the daily rest of 11 h.”

Shortening weekly rest limit was a single-item measure. “Fill in the number of times you shortened the weekly rest of 45 h in the last month.”

### 3.3. Procedure

The two-step approach as suggested by Anderson and Gerbing [34] was followed with the first evaluating measurement model via exploratory and confirmatory factor analysis and second building structural equation model. Robust maximum likelihood method of parameter estimation as proposed by Boomsma and Hoogland [35] for data not following multivariate normal distribution was used for evaluation of measurement and structural model. The error variance of the constructs with a single indicator was set to 0.

The signs of convergent validity were statistically significant and considerable (>0.50) loadings of items on the factor, they were supposed to measure [34,36] and good overall fit of the model. Composite reliability above 0.60 was considered an indicator of good reliability and average variance extracted (AVE) above 0.50 an indicator of good construct validity [37]. Reliability was assessed also by Cronbach’s α. The values above 0.70 were considered to indicate adequate reliability as proposed by Nunnally [38] and the value above 0.60 as a still acceptable level of reliability [39]. Discriminant validity was assessed by 95% confidence interval for correlation coefficients between each pair of constructs. Confidence interval should not include 1 [40] and by AVE, which should not be higher than the squared correlation between the latent variables [37]. After establishing good measurement validity, structural equation model was built. The fit of the model was evaluated using the Sattora–Bentler scaled Chi Square, which is suitable for evaluating models with non-normal data [35]. In addition, the Comparative Fit Index (CFI), Incremental fit index (IFI), non-normed fit index (NNFI), the Root Mean Square Error of Approximation (RMSEA), and the Standardized Root Mean Square Residual (SRMR) were used. Values of 0.95 or and above or 0.90 and above for CFI, NNFI, and IFI, and values of 0.08 and below for RMSEA and SRMR indicate a good fit of the model [41]. LISREL 9.30 (Vector Psychometric Group llc, Chapel Hill, NC, USA) was used for model calibration and hypotheses testing.

## 4. Results

Confirmatory factor analysis resulted in good overall fit of the measurement model (SB χ^2^ = 168.8; df = 134; *p* = 0.02; SB χ^2^/df = 1.3; RMSEA = 0.03; NFI = 0.94; NNFI = 0.98; CFI = 0.99; IFI = 0.99; SRMR = 0.05). Standardized loadings, AVE and composite reliability measures for multi-item factors are summarized in Table 1. All factor loadings are higher than 0.50 and statistically significant. Together with the overall good fit of the model, they are indicators of convergent validity. Further construct validity is supported by AVE above 0.50 for the majority of constructs. Only construct measuring financial incentives is a bit below the proposed threshold, but all loadings are above recommended 0.50 and are statistically significant. Composite reliability for all constructs is above the 0.70 threshold, indicating sufficient measurement reliability.

Correlation coefficients between constructs are rather low with the highest value of r = 0.51 (Table 2). None of the 95% confidence intervals for correlation coefficient includes 1 and none of the AVE is higher than the squared correlation coefficient between the pair of constructs. All these suggest that constructs differ from one another and support the discriminant validity of measurement.

**Figure 2:**
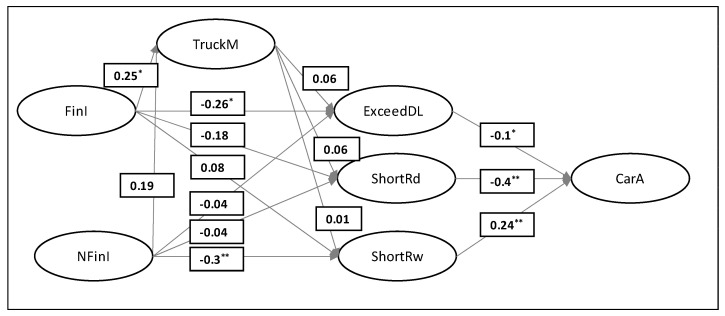
Structural equation model path diagram (standardized path coefficients are shown). FinI = financial incentives; TruckM = truck management; CarA = car accidents; NfinI = non-financial incentives; ExceedDL = Exceeding daily driving limit; ShortRd = shortening daily rest limit; ShortRw = shortening weekly rest limit; * *p* < 0.05; ** *p* < 0.01.

Structural equation model exhibited good overall fit (SB χ^2^ = 211.4; df = 140; *p* < 0.001; SB χ^2^/df = 1.51; RMSEA = 0.05; NFI = 0.92; NNFI = 0.97; CFI = 0.97; IFI = 0.97; SRMR = 0.073). The path diagram with standardized coefficients is depicted in Figure 2. Six out of 14 hypotheses are supported by the data. Financial incentives negatively impact the exceeding of the daily driving limit. Non-financial incentives negatively impact shortening daily rest. Financial incentives are positively related to the drivers’ ability of truck management. The number of car accidents is influenced by drivers’ frequent exceeding of daily driving limit, shortening of daily, and weekly rest.

## 5. Discussion

The results show that higher financial incentives reduce the propensity of truck drivers to exceed driving time limits and are in line with studies conducted in the US and Australia, where it has been shown that better-paid drivers are also more safety-oriented [1,4,42,43]. They also confirm the findings of Belzer and Sedo [5], who found that truck drivers in the USA with higher compensation reach their target earnings sooner and will therefore work fewer kilometers and fewer hours, which will reduce the need to violate working hours rules. Financial incentives do not affect the shortening of daily or weekly rest. Shortening the weekly rest is especially problematic, as drivers who have engaged in this practice have also caused more traffic accidents. Interestingly, drivers who have exceeded the daily driving limit on several occasions actually caused fewer traffic accidents throughout their lifetime. Murphy et al. [26,27] provide an explanation for this phenomenon. The authors point out that through their work experience as truck drivers, which includes their exposure to social and external systems, both in a narrow (e.g., specific maintenance regulations) and wide sense (e.g., national norms or culture), they have developed their own beliefs that help them do their job with efficiency. More experienced drivers will be better at distributing their work tasks throughout the day in order to get enough sleep, which is why violations of hours of service regulations will not have a significant effect on their fatigue levels and consequently will not expose them to a higher probability of traffic accidents. Here, a connection can be made to Faulkiner and Belzer [4], who found that experienced drivers had lower average crash costs and were more productive.

The results showed a positive impact of non-financial work incentives on weekly rest shortening, as drivers who receive better non-financial motivation are also less likely to shorten their weekly rest. This effect of non-financial work incentives can also be attributed to the results of a survey on the subjective well-being of employees in the transport sector, which revealed that the data in this sector is well below average in terms of life satisfaction and job satisfaction [17]. According to Stewart et al. [18], the well-being of workers has a significant effect on work productivity, and non-financial work incentives create the basis for greater work motivation.

Higher financial incentives affect the ability of truck drivers to perform their job, as results showed that drivers still feel fit to drive after they have reached their daily driving time limit. The same applies to rest, as drivers fell that they could have started driving again even sooner, at the end of their daily rest period. In addition to that, truck drivers feel rested when they are finishing their workweek on Fridays, which means that they are able to effectively balance their work obligations during the week with adequate rest and other activities that contribute to less fatigue and better driving abilities. These drivers also have no propensity for exceeding the daily driving time or shortening the daily or weekly rest period. To some extent, these results are consistent with efficiency wage theory, which is based on the assumption that offering higher compensation attracts more capable and motivated job candidates [4,5]. Becker [23] and Kudo and Belzer [1] also point out that workers with greater human capital can be more productive and possess high-level work skills, allowing companies to pay them higher financial compensation.

The results of the SEM model provide answers to the research questions. Contrary to our assumption that a satisfied or financially better-compensated truck driver might, in certain situations, be even more inclined to commit movement violations, the results were consistent with studies conducted over the past five years. Financial incentives do have a significant impact on the safety behavior of truck drivers, which is a direct answer to the first research question. However, the fact remains that in the current situation in the transport sector, higher pay is often associated with a higher number of kilometers driven. Drivers who drive more kilometers are more likely to be exposed to greater risk [25,44]. The authors also mention poor working conditions, which is why the second research question examined whether non-financial compensation of truck drivers could be an important factor in ensuring safe working conditions in the transport sector. The results showed that the higher the non-financial incentives, the less likely were drivers to shorten their weekly rest period over the last month. Here, we should point out that shortening the weekly rest period is a key cause of road accidents. That would suggest that higher non-financial incentives result in fewer cases of shortening the weekly rest period and thus fewer road accidents. This answers the second research question that proposes new operational guidelines in the transport sector. The importance of ensuring non-financial job properties has also been recognized by the European Parliament, with the adoption of a mobility package in July 2020, which will provide better working conditions for truck drivers [45]. The results related to the third research question also offer clear answers and to some extent confirm the current situation in the transport sector. Higher financial incentives do affect the ability of truck drivers to do their job. These workers tend to be more productive and can possess high-level work skills, which allows companies to pay them higher financial compensation [1,4].

### 5.1. Contribution to Theory and Practice

The results of the study represent a scientific novelty, as no similar study has been conducted in the EU to address the impact of compensation on the safety behavior of truck drivers. This lack of research is surprising, given the size and importance of the transport sector in the area. The results build on the findings of previous studies that address the importance of financial incentives [4,5,6,13,19,44,46] and non-financial incentives [16]. A contribution is made to the following areas of compensation in the transport sector:

- Legislative improvements in the EU: The results provide an important basis for decision-makers at the level of the EU, as there is a clear and pressing need to redefine the compensation of truck drivers and recognize the important role of non-financial incentives in improving the situation in the transport sector. The need to improve the situation in the transport sector at the level of the European Community was also recognized at the European Parliament’s plenary session on 8 July 2020 through the confirmation of the mobility package. The new rules will provide a clear legal framework to prevent differing national approaches and ensure better working conditions for drivers, fairer competition, and fighting against illegal practices, as well as ensuring equal and fair pay for all truck drivers in the EU [45]. The results of the study confirm that there is a need for such new directions.
- Improvements to non-financial compensation in transport companies: The results show that non-financial work incentives have an impact on drivers’ safety behavior. This represents an upgrade of the study conducted by Prockl et al. [16] who found that the provision of non-financial incentives in transport companies results in increased loyalty towards the employer. Providing non-financial job properties will not only ensure increased loyalty towards the employers but also encourage better safety behavior among truck drivers. In the long run, this will also reduce costs stemming from road accidents and fines for traffic offences. Companies will have fewer loss events, which also means lower insurance premiums, better reputation, and better quality transport services [42].
- Improvements to financial compensation of truck drivers: The results prove that increased financial compensation does have an impact on the safety behavior of truck drivers and confirm previous research findings. Thus, the results are indicative of the ongoing initiatives of researchers to introduce “safe rates”. Belzer and Sedo [5] suggest that a safe mileage rate, in the United States, would be around 60 cents per mile, which is approximately 50% greater than the current average rate. In Australia, the Transport Workers’ Union [47] has been running a comprehensive “safe rates” campaign for the past several years, with the goal of raising standards for all carriers. They point out that road transport is Australia’s deadliest industry. When wealthy companies at the top of supply chains squeeze transport contracts, the rest of the supply chain is pressured to cut corners in safety. Transport workers are forced to work longer and harder to make ends meet. Far too many people are killed in truck crashes every year, while their families and communities are left devastated [47]. In response to the interest of the International Trade Union Confederation, the International TWU, the International Organization of Employers and the International Road Transport Union (ILO), the International Labor Organization (ILO) hosted a tripartite sectoral council meeting on road safety and health, published a report [48] and issued a resolution indicating the need for safe rates, based on the discussions [49,50,51]. At the moment, however, these initiatives are hindered by a structural problem in the EU, which is mainly due to the significant differences in the level of financial compensation between the richer western part of the EU and the newer members. Despite the fact that the European Parliament has adopted guidelines in the new legal framework that ensure equal and fair pay for all truck drivers in the EU, the question remains as to how the financial compensation of drivers will be systematically regulated. The reality of the situation at the global level and the results of the research clearly indicate that the legislative introduction of »safe rates« will play an important role in ensuring workplace safety and resolving the social issues that exist in the transport sector.

Our final proposal for improving the situation in the transport sector focuses on resolving two structural issues that not only affect the transport sector in the EU but also in the rest of the world. The first proposal, which is to pay drivers at a statutory hourly rate, is also highlighted by other authors in the USA [1,5] and in Brazil [46]. The issue is that most drivers do a lot of unpaid work besides driving or spend many hours waiting for the truck to be loaded or unloaded. If the law required drivers to be compensated for such tasks, it would be in everyone’s interest to improve the supply chain work system. This would result in less time spent waiting for various logistics processes such as preparing documentation or moving goods to be finished. In the long run, this would benefit everyone, as work being done faster within logistics processes could reduce costs. Truck drivers would be compensated fairly for all the other task that they perform, even those that do not involve driving. This would have a tangible impact on road safety and, last but not least, on the health of the drivers. This, in turn, creates the basis for resolving another structural problem that relates to the elimination of deregulation of the truck driver profession. The fact is that virtually anyone who obtains a driving license and a code 95 driver qualification can become employed as a truck driver [52,53]. As for education, a secondary-level education is sufficient, even if uncompleted. One must take into account that driving a 40-ton truck in international transport is a demanding job that requires a certain level of responsibility, at least basic conversational proficiency in a foreign language, communication skills, knowledge of administrative procedures, being able to take good care of the cargo and some technical knowledge about the inner workings of a truck. Better working conditions that do not depend on the number of kilometers driven, and fair pay would attract quality job candidates to the sector, and the truck driver profession would once again become a respected and attractive profession.

### 5.2. Limitations and Recommendations for Future Studies

This is the first study that deals with the subject of the compensation of truck drivers in the EU. Therefore, it was not possible to make a substantial comparison of the results with previous studies. There was only one similar study that was conducted on a sample of 143 truck drivers employed by German companies and addressed the antecedents of truck drivers’ job satisfaction and retention proneness [16]. The results of our study reflect the situation in the EU transport sector in the past year. On 8 July 2020, the European Parliament adopted the mobility package during its plenary session. The package provides new guidelines for operations in the road transport sector, which suggest that the study should be repeated at a later time, to see whether the initiatives aimed at regulating working and safety conditions have achieved their purpose [45]. In this respect, the limitations of the study also suggest recommendations for future research.

The next recommendation is related to future advancements in terms of truck technology, driver assistance systems and the transition to automated driving. The role of the truck driver will change drastically in the future, as the demand for drivers will decrease. However, some truck drivers will remain indispensable, which will require researchers and lawmakers to keep up with new developments and redefine both the work competencies and the compensation system for truck drivers.

## 6. Conclusions

At the global level, a lot of research has been conducted on the subject of truck driver compensation, but this is the first study to address this issue in the EU. The obtained results represent an upgrade of the current field of research, as they point out that in the current state of the transport industry, financial and non-financial incentives have a positive impact on the work, social, and safety behavior of drivers. Financial incentives also have an impact on drivers’ increased perception of their driving ability, while moving violations continue to have a major impact on the number of accidents. The results of the SEM model offer answers to the research questions and provide decision-makers at the highest level with a basis for the adoption of regulative instruments that will help resolve the issues that have been affecting the industry from a work, social and safety point of view for the past several years. A contribution is made to the following areas in the transport sector: legislative improvements in the EU, improvements to non-financial compensation in transport companies and improvements to financial compensation of truck drivers.

This is the first study that deals with the subject of the compensation of truck drivers in the EU. Therefore, it was not possible to make a substantial comparison of the results with previous studies. To address the long-standing issues in the transport industry, on 8 July 2020, the European Parliament adopted new guidelines, which will include better working conditions for drivers, fairer competition, and ensure fair pay for all truck drivers. Therefore, it would be advisable to repeat the study after a certain period of time. Repeating the study would confirm whether the initiatives introduced to improve working and safety conditions in the industry have achieved their purpose.

## Figures and Tables

**Table 1:** Standardized loadings, average variance extracted (AVE) and composite reliability (CR) for multi-item constructs (results of confirmatory factor analysis).

Financial Incentives (CR = 0.77; AVE = 0.47)	Std. Loading
The salary is sufficient for covering living expenses.	0.56
Working on a free weekend outside home results in additional financial incentives.	0.65
Working on holidays results in additional financial incentives.	0.86
Spending less fuel than normative results in additional financial incentives.	0.62
Non-financial incentives (CR = 0.89; AVE = 0.59)	
The communication with my superior is correct.	0.75
Drivers in the company help each other.	0.61
In the company, they praise me when I deserve it.	0.79
When I started working, the company helped me with inclusion into the working process.	0.70
In the company, they are ready to help me if I need something.	0.83
The company superiors respect me.	0.88
Truck management (CR = 0.75; AVE = 0.51)	
When the daily time of driving 9/10 h is ending, I still feel capable of driving.	0.78
When the daily rest time of 11 h is ending, I feel able to start driving sooner.	0.76
On Fridays, at the end of the workweek, I feel rested.	0.58
Car accidents (CR = 0.79; AVE = 0.56)	
Fill in the number of accidents with financial damage.	0.81
Fill in the number of accidents where one or more people suffered minor injuries.	0.69
Fill in the number of accidents where one or more people suffered major injuries.	0.74

**Table 2:** Correlations between factors.

Factors	FinI	TruckM	CarA	NfinI	ExceedDL	ShortRd	ShortRw
FinI	1.00						
TruckM	0.35 ***	1.00					
CarA	0.28 ***	0.11	1.00				
NfinI	0.51 ***	0.31 ***	0.06	1.00			
ExceedDL	−0.26 ***	−0.04	−0.13	−0.14 *	1.00		
ShortRd	−0.17 *	−0.02	−0.36 ***	−0.10	0.22 **	1.00	
ShortRw	−0.04	−0.07	0.10	−0.25 ***	0.28 ***	0.30 ***	1.00

FinI = financial incentives; TruckM = truck management; CarA = car accidents; NfinI = non-financial incentives; ExceedDL = Exceeding daily driving limit; ShortRd = shortening daily rest limit; ShortRw = shortening weekly rest limit; * *p* < 0.05: ** *p* < 0.01; *** *p* < 0.001.
